# Inhalable Metal-Rich Air Particles and Histone H3K4 Dimethylation and H3K9 Acetylation in a Cross-sectional Study of Steel Workers

**DOI:** 10.1289/ehp.1002955

**Published:** 2011-03-08

**Authors:** Laura Cantone, Francesco Nordio, Lifang Hou, Pietro Apostoli, Matteo Bonzini, Letizia Tarantini, Laura Angelici, Valentina Bollati, Antonella Zanobetti, Joel Schwartz, Pier A. Bertazzi, Andrea Baccarelli

**Affiliations:** 1 Department of Environmental and Occupational Health, Università di Milano and Istituto Di Ricovero e Cura a Carattere Scientifico, and Maggiore Hospital, Mangiagalli and Regina Elena Foundation, Milan, Italy; 2 Department of Clinical Medicine, Nephrology and Health Sciences, University of Parma Medical School, Parma, Italy; 3 Department of Preventive Medicine, Feinberg School of Medicine, Northwestern University, Chicago, Illinois, USA; 4 Department of Experimental and Applied Medicine, Occupational Medicine and Industrial Hygiene, University of Brescia, Brescia, Italy; 5 Department of Clinical and Biological Sciences, University of Insubria, Varese, Italy; 6 Laboratory of Environmental Epigenetics, Exposure Epidemiology and Risk Program, Department of Environmental Health, Harvard School of Public Health, Boston, Massachusetts, USA

**Keywords:** environmental carcinogens, epigenetics, histone modifications, metals, particulate matter

## Abstract

Background: Epidemiology investigations have linked exposure to ambient and occupational air particulate matter (PM) with increased risk of lung cancer. PM contains carcinogenic and toxic metals, including arsenic and nickel, which have been shown in *in vitro* studies to induce histone modifications that activate gene expression by inducing open-chromatin states. Whether inhalation of metal components of PM induces histone modifications in human subjects is undetermined.

Objectives: We investigated whether the metal components of PM determined activating histone modifications in 63 steel workers with well-characterized exposure to metal-rich PM.

Methods: We determined histone 3 lysine 4 dimethylation (H3K4me2) and histone 3 lysine 9 acetylation (H3K9ac) on histones from blood leukocytes. Exposure to inhalable metal components (aluminum, manganese, nickel, zinc, arsenic, lead, iron) and to total PM was estimated for each study subject.

Results: Both H3K4me2 and H3K9ac increased in association with years of employment in the plant (*p*-trend = 0.04 and 0.006, respectively). H3K4me2 increased in association with air levels of nickel [β = 0.16; 95% confidence interval (CI), 0.03–0.3], arsenic (β = 0.16; 95% CI, 0.02–0.3), and iron (β = 0.14; 95% CI, 0.01–0.26). H3K9ac showed nonsignificant positive associations with air levels of nickel (β = 0.24; 95% CI, –0.02 to 0.51), arsenic (β = 0.21; 95% CI, –0.06 to 0.48), and iron (β = 0.22; 95% CI, –0.03 to 0.47). Cumulative exposures to nickel and arsenic, defined as the product of years of employment by metal air levels, were positively correlated with both H3K4me2 (nickel: β = 0.16; 95% CI, 0.01–0.3; arsenic: β = 0.16; 95% CI, 0.03–0.29) and H3K9ac (nickel: β = 0.27; 95% CI, 0.01–0.54; arsenic: β = 0.28; 95% CI, 0.04–0.51).

Conclusions: Our results indicate histone modifications as a novel epigenetic mechanism induced in human subjects by long-term exposure to inhalable nickel and arsenic.

Ambient and occupational exposure to inhalable particulate matter (PM) has been associated with increased risk of lung cancer (Dockery et al. 1993; Gibb et al. 2000;Kuo et al. 1999). Epidemiologic and *in vivo* studies suggest that the metal components of PM may be responsible for PM health effects, including lung cancer (Chang et al. 2005; Chen and Hwang 2005; Conroy et al. 2008; Corey et al. 2006; Coyle et al. 2006; Franklin et al. 2008; MacNee and Donaldson 2003; Roller 2009; Wild et al. 2009). Despite the well-recognized carcinogenic potentials of several toxic metals, the molecular mechanisms underlying their associations with cancer risk remain poorly understood. In particular, most carcinogenic metals are weak mutagens and do not induce DNA adduct formation, a key initiating event caused by other carcinogens (Salnikow and Zhitkovich 2008).

Growing evidence indicates that epigenetic dysregulation of gene expression plays a primary role in cancer etiology (Feinberg and Tycko 2004; Ke et al. 2006). Several toxic metals have been shown to bind more avidly to histone proteins than to other biopolymers such as DNA or RNA (Conroy et al. 2008; Zoroddu et al. 2000, 2002). Recent *in vitro* studies have shown that carcinogenic metals cause posttranslational epigenetic modifications of histone proteins, thus derailing the normal programming of gene expression (Ke et al. 2006; Yan and Boyd 2006; Yan et al. 2003; Zhou X et al. 2009). In lung epithelial cell lines and malignant transformation models, arsenic (Jensen et al. 2009; Zhou X et al. 2009), nickel (Zhou QX et al. 2009), and chromium (Sun et al. 2009; Zhou X et al. 2009) have been linked with specific activating histone modifications, such as H3K4 (histone 3 lysine 4) dimethylation (H3K4me2), that contribute to the formation of a relaxed or “open” chromatin structure permissive for gene transcription (Surralles et al. 1998; Yan et al. 2003). Metal-related induction of activating histone modifications have been suggested to contribute to metal carcinogenesis by causing the expression of tumor suppressor or other cancer-promoting genes (Wang et al. 2005; Zhou X et al. 2008). Inhaled PM pollutants have been previously shown to produce systemic changes in gene expression, which can be detected in peripheral blood of exposed individuals (Salnikow and Zhitkovich 2008). However, whether metals in inhalable PM induce alterations of histone modifications in human subjects has never been evaluated.

Although wide strata of the general population are exposed to low, background levels of toxic and carcinogenic inhalable compounds, industrial workers have been often exposed to significantly higher concentrations of potentially carcinogenic agents released from multiple sources (MacArthur et al. 2009). Foundry work is a specific condition of exposure to inhalable metal-rich PM that has been associated with increased risk of lung cancer in several early investigations (International Agency for Research on Cancer 1987). Even in modern foundry facilities, PM exhibits levels that are well above the concentrations found in ambient outdoor air and also have a larger proportion of toxic metal components (Alley et al. 2009; Fang et al. 2009; Roy et al. 2009; Vijay Bhaskar et al. 2009). In the present investigation in a steel furnace plant, we investigated whether metals in inhalable PM determined changes in H3K4me2 and histone 3 lysine 9 acetylation (H3K9ac), two histone modifications associated with open chromatin states, in peripheral blood leukocytes from workers with well-characterized exposure.

## Materials and Methods

*Study subjects.* We recruited 63 male healthy workers, free of cancer and cardiopulmonary disease, who had been working in a steel production plant in Brescia, Italy, for at least 1 year. Individual written informed consent and approval from the local institutional review board were obtained before the study. All of the study subjects had a rotating weekly schedule based on 4 consecutive working days of 8 hr each, followed by 2 days of rest. The study subjects worked in 11 different areas within the plant, which were selected to provide a wide contrast of exposures between the study subjects. The exposure of each of the study subjects in the plant was monitored for the first 3 working days of a work week. On the fourth day, each subject donated a 20-mL peripheral blood sample. We used ethylene diamine tetraacetic acid (EDTA) tubes to collect 7 mL whole blood that was promptly (within 30 min from the blood drawing) centrifuged on site at 2,500 rpm for 15 min. The buffy coat (400 μL) was separated and transferred in a cryovial, immediately snap frozen in vapor phase of liquid nitrogen, shipped in nitrogen dry shippers to the laboratory, and kept stored in vapor phase of liquid nitrogen until histone extraction. A self-administered questionnaire was used to collect detailed information on lifestyle, drug use, medical conditions, body mass index (BMI), education, and residential history. Records from the factory administrative files were used to extract information on occupational history.

*Exposure assessment.* PM metal components (aluminum, manganese, nickel, zinc, arsenic, lead, iron) and PM mass [PM with aerodynamic diameters ≤ 10 μm and ≤ 1 μm (PM_10_ and PM_1_, respectively)] were measured in each of the 11 work areas of the steel production plant. We measured air concentrations of individual metal PM components in PM_10_, through multielement analysis performed by means of inductively coupled-plasma mass spectrometer (ELAN DRC II; PerkinElmer, Waltham, MA, USA) using the total quant method. External calibration was performed using calibration standard 3, stock multielement (10 μg/mL; PerkinElmer). PM_10_ and PM_1_ were measured using a Grimm 1100 light-scattering dust analyzer (Grimm Technologies, Inc., Douglasville, GA, USA).

Study subjects recorded in a personal log the time they spent in each of the work areas. Personal exposure was calculated as the average of work area levels weighted by the time spent in each area. All metal and PM levels were expressed in micrograms per cubic meter. In the plant, exposure levels have shown very little variability over time, as measures repeated over 3 years in a subset of the study population showed very high correlations (*r*^2^ > 0.90). Therefore, the time-weighted levels of metals and PM represented, in addition to the exposure during the week of the study, also a measure of the usual exposure of the study subjects (Tarantini et al. 2009). We estimated cumulative exposures as the product of the time-weighted levels of metals and PM during the study by the years of employment in the plant.

*Total histone extraction and histone modification analysis.* We obtained buffy coat from peripheral blood collected in EDTA tubes centrifuged at room temperature (22–25°C) for 15 min at 1,500 × *g*. Red blood cell lysis solution (s.r.l. cod.A7933; Promega, Madison, WI, USA) was added to the buffy coat to wash out red blood cells. After 10 min at room temperature, the mixture was centrifuged at 2,500 × *g* for 15 min, and the supernatant was discarded. Remaining monolayer cells were processed according to protocol used by Chen et al. (2006). Briefly, cells were lysed in 1 mL ice-cold radioimmunoprecipitation assay buffer (Santa Cruz Biotechnology, Santa Cruz, CA, USA) supplemented with a protease inhibitor mixture (Roche Applied Sciences, Indianapolis, IN, USA) for 10 min. The sample was then collected and centrifuged at 10,000 × *g* for 10 min. After discarding supernatant, remaining pellet was resuspended in 0.4 N H_2_SO_4_. After incubation on ice for 90 min, the sample was centrifuged at 14,000 × *g* for 15 min. The supernatant was mixed with cold acetone and kept at –20°C overnight. The histones were collected by centrifugation at 14,000 × *g* for 15 min. After one wash with acetone, the histones were air dried and resuspended in 500 μL water. We measured total proteins in each sample by means of the Bradford assay according to manufacturer’s instructions (protein assay kit 500-0002; Bio-Rad Laboratories, Milan, Italy). We used equal amounts of proteins (4 μg) to normalize results of the subsequent analysis on histones.

We used a solid-phase sandwich enzyme-linked immunosorbent assay (ELISA), using monoclonal antibodies, to detect endogenous levels of H3K4me2 and H3K9ac (PathScan Sandwich ELISA Kits; Cell Signaling Technology, Beverly, MA, USA) according to the manufacturer’s protocol. The assays we used in our study are the PathScan dimethyl-histone H3 (Lys4) Sandwich ELISA Kit 7124 and PathScan acetyl-histone H3 (Lys9) Sandwich ELISA Kit 7121. The assays use dimethyl-histone H3 (Lys4) (C64G9) rabbit monoclonal antibody 9725 and acetyl-histone H3 (Lys9) antibody 9671, respectively, which have been shown by the manufacturer to be specific for the histone modifications of concern. Sample measurements were performed in duplicate. We used a Synergy HT-BioTek spectrophotometer (Winooski, VT, USA) to read 450 nm absorbance. The absorbance values at 450 nm directly reflected the concentration of modified histones (Deligezer et al. 2010). According to the Beer–Lambert law, optical density (OD; absorbance) is used for colorimetric analysis so that readings relate directly to concentration. The coefficient of variation in replicate samples of the assays was 0.30% for H3K4me2 and 0.42% for H3K9ac.

*Statistical analysis.* We performed trend tests using the continuous variable in the regression model and presenting the corresponding two-sided *p*-values. We evaluated the association of PM metal components and PM mass levels with histone modifications using simple linear regression models, as well as multivariable models adjusting for age, BMI, pack-years, and percent granulocytes in the differential blood count as continuous variables and education as categorical variable. The independent variables used in multivariable models were selected *a priori* and included general characteristics potentially associated with cancer risks or other carcinogenic exposures. In addition, we adjusted for percent granulocytes to account for possible shifts in the proportion of leukocytes subtypes associated with the exposures. As sensitivity analyses, we fitted in the models as independent variables data from differential white blood counts (i.e., percent lymphocytes, monocytes, eosinophils, or basophils) or duration of sample storage and found no major changes in the results. Regression diagnostics were performed separately for each model. We examined whether the exposure–response relationships were linear through graphical inspection. Furthermore, we fitted a polynomial regression by including a quadratic term for exposure and compared these models with the linear model using the likelihood ratio test. Neither graphical inspection nor the likelihood ratio tests suggested any departure from linearity.

Outliers were excluded from regression analysis by dropping observations with studentized residuals that exceeded +3 or –3. The number of outliers removed ranged from a minimum of 0 to a maximum of 3.

Regression coefficients were computed with ordinary least squares estimators. To compare the magnitude of the associations of H3K4me2 and H3K9ac with different exposures, we calculated standardized regression coefficients and 95% confidence intervals (CIs) that express the change in histone modifications associated with an increase in exposure equal to the difference between the 90th and 10th percentile of the exposure distribution. We checked regression assumptions by performing diagnostic tests for each model, which included the Shapiro–Wilk test to verify normality of residuals and the White test to verify the homogeneity of variance of the residuals. A two-sided *p*-value < 0.05 was considered statistically significant. Statistical analyses were performed in SAS (version 9.1.3; SAS Institute Inc., Cary, NC, USA) and R (R Foundation for Statistical Computing, Vienna, Austria).

## Results

*Subjects’ characteristics and exposure levels.* The mean age of the study subjects was 44.00 years (range, 27–55 years). Twenty-five subjects (40%) were current smokers, who reported a median number of 15 cigarettes smoked every day (range, 0–30 cigarettes/day). The BMI of the study participants showed a median of 26 kg/m^2^ (range, 20–33 kg/m^2^). Table 1 shows the average levels of inhalable air metal concentrations and PM mass estimated. For both metal levels and PM mass, the study subjects showed wide ranges of exposures. The subject with the maximum individual exposure level was at least 17 times more exposed than the subject with the minimum exposure level (e.g., for PM_10_: maximum, 1220.17 μg/m^3^; minimum, 73.72 μg/m^3^). For some of the exposures (aluminum, manganese, zinc, lead), the maximum individual exposure was > 200 times higher than the minimum individual exposure (Table 1).

**Table 1:** Levels of personal exposure to metal components of PM and total PM mass.

								Percentile						
Exposure*a* (μg/m^3^)		*n*		Mean ± SD		Minimum		25th		50th		75th		Maximum
Aluminum		63		8.50 ± 18.07		0.40		1.48		2.05		7.41		84.07
Manganese		63		11.26 ± 30.41		0.11		1.20		4.63		10.77		174.79
Nickel		63		0.30 ± 0.18		0.02		0.23		0.25		0.46		0.72
Zinc		63		18.85 ± 26.37		0.26		1.47		8.45		32.28		129.06
Arsenic		63		0.10 ± 0.1		0.01		0.02		0.07		0.17		0.31
Lead		63		7.53 ± 17.46		0.13		0.63		2.87		9.52		99.90
Iron		63		32.02 ± 22.08		0.96		18.00		25.64		48.69		88.43
PM_10_		63		233.42 ± 214.56		73.72		152.23		179.45		222.86		1220.17
PM_1_		63		8.48 ± 6.18		1.71		3.51		9.01		11.35		30.49
**a**Metal components were measured on the PM_10_ fraction of PM mass. Coarse PM levels were calculated as the difference between PM_10_ and PM_1_.														

The correlation matrix between exposure levels showed correlations of different strengths between the exposures (Table 2). We found high correlations [Pearson’s correlation coefficient (*r*) = 0.7–1] for aluminum with manganese, lead, and PM_10_; for manganese with lead and PM_10_; for nickel with arsenic and iron; and between arsenic and iron, PM_10_ and lead, and PM_1_ and PM_10._ Correlations were moderate (*r* = 0.4–0.7) for PM_1_ with aluminum, manganese, and lead, between nickel and aluminum, and between iron and zinc. Except for PM_1_ and arsenic (*r* = –0.20), all other correlations were positive with *r* < 0.40.

**Table 2:** Matrix of correlations (*r*)*a* among individual exposures (PM metal components and PM mass measures).

Exposure		Aluminum		Manganese		Nickel		Zinc		Arsenic		Lead		Iron		PM_10_		PM_1_
Aluminum		1																
Manganese		0.75		1														
		< 0.0001																
Nickel		0.46		0.39		1												
		0.0001		0.0017														
Zinc		0.18		0.21		0.32		1										
		0.1625		0.1034		0.0105												
Arsenic		0.15		0.31		0.84		0.25		1								
		0.2378		0.0143		0.0000		0.0483										
Lead		0.75		0.99		0.38		0.35		0.28		1						
		< 0.0001		< 0.0001		0.0018		0.0047		0.0265								
Iron		0.15		0.28		0.76		0.48		0.70		0.32		1				
		0.2451		0.0261		< 0.0001		0.0001		< 0.0001		0.0113						
PM_10_		0.81		0.82		0.34		0.34		0.04		0.85		0.27		1		
		< 0.0001		< 0.0001		0.0056		0.0070		0.7446		< 0.0001		0.0294				
PM_1_		0.63		0.60		0.22		0.28		–0.20		0.64		0.26		0.90		1
		< 0.0001		< 0.0001		0.0897		0.0277		0.1195		< 0.0001		0.0408		< 0.0001		
**a**Pearson’s product-moment correlation coefficient and corresponding *p*-values.																		

*Associations of histone modifications with subjects’ characteristics and years of employment.* The range of values of the histone modifications was 0.26–1.20 OD for H3K4me2 (median, 0.99 OD) and –0.50 to 1.06 OD for H3K9ac (median, 0.37 OD).

H3K4me2 was moderately but significantly correlated with H3K9ac (Pearson’s *r* = 0.45, *p* < 0.001). H3K4me2 and H3K9ac were not associated with age, BMI, smoking, number of cigarettes/days, smoking duration, pack-years, area of residence (city center, suburbs, rural), self-reported traffic intensity near home, or percent granulocytes (Table 3). H3K9ac was positively associated with education (*p* = 0.04; Table 3). Both H3K4me2 and H3K9ac increased in association with the years of employment of the study subjects in the steel plant (Table 3). We confirmed the association between years of employment and histone modifications in age-adjusted regression models.

**Table 3:** Association of the subjects’ characteristics with H3K4me2 and H3K9ac (in OD units) measured on the fourth day of a work week.

				H3K4me2			H3K9ac							H3K4me2			H3K9ac	
Variable		*n*		Mean OD (95% CI)	*p*-Value*a*		Mean OD (95% CI)	*p*-Value*a*		Variable		*n*		Mean OD (95% CI)	*p*-Value*a*		Mean OD (95% CI)	*p*-Value*a*
Age (years)										Education								
< 39		22		0.90 (0.79–1.02)			0.41 (0.22–0.59)			Primary school		12		0.96 (0.83–1.09)			0.25 (0.05–0.45)	
39–47		20		1.00 (0.96–1.05)			0.43 (0.26–0.59)			Middle school		37		0.94 (0.89–0.99)			0.43 (0.31–0.56)	
> 47		21		0.94 (0.88–1.00)	0.47		0.43 (0.27–0.58)	0.88		High school		14		0.96 (0.81–1.10)	0.98		0.55 (0.36–0.74)	0.04
BMI (kg/m^2^)										Area of residence								
< 25		21		0.96 (0.9–1.02)			0.48 (0.33–0.62)			City center		8		0.89 (0.54–1.23)			0.30 (0.05–0.64)	
25–27.5		21		0.89 (0.78–1.01)			0.20 (0.04–0.35)			Suburbs		41		0.97 (0.92–1.01)			0.49 (0.38–0.60)	
> 27.5		21		0.99 (0.95–1.03)	0.57		0.59 (0.45–0.74)	0.36		Rural		12		0.91 (0.79–1.03)	0.94		0.26 (0.02–0.50)	0.43
Smoking										Self-reported traffic intensity near home								
Nonsmoker		24		0.96 (0.88–1.04)			0.44 (0.28–0.61)			High		5		0.86 (0.43–1.29)			0.39 (0.15–0.92)	
Former smoker		14		0.87 (0.76–0.97)			0.35 (0.12–0.59)			Medium		38		0.94 (0.89–1.00)			0.46 (0.33–0.58)	
Current smoker		25		0.98 (0.92–1.05)	0.14*b*		0.43 (0.30–0.57)	0.76*b*		Low		18		0.97 (0.91–1.03)	0.26		0.36 (0.18–0.53)	0.57
No. of cigarettes/day										Granulocyte (%)								
0		38		0.93 (0.87–0.99)			0.41 (0.28–0.54)			45–55		21		0.95 (0.88–1.01)			0.43 (0.3–0.57)	
1–10		10		0.98 (0.92–1.05)			0.39 (0.23–0.55)			55–61		21		0.94 (0.82–1.06)			0.42 (0.19–0.65)	
> 10		15		0.98 (0.87–1.09)	0.29		0.46 (0.24–0.68)	0.69		61–76		21		0.96 (0.91–1.02)	0.76		0.41 (0.28–0.54)	0.86
Smoking duration (years)										Years of employment								
0		23		0.97 (0.88–1.05)			0.45 (0.28–0.62)			< 9		20		0.88 (0.75–1.00)			0.22 (0.04–0.39)	
0–19		19		0.90 (0.8–1.00)			0.35 (0.15–0.54)			9–21		17		0.96 (0.91–1.02)			0.43 (0.28–0.58)	
> 19		21		0.97 (0.91–1.03)	0.94		0.45 (0.32–0.58)	0.98		> 21		22		0.99 (0.95–1.04)	0.04		0.51 (0.37–0.66)	0.006
Pack-years																		
0		24		0.96 (0.88–1.04)			0.44 (0.28–0.61)											
0–4		17		0.92 (0.84–0.99)			0.39 (0.22–0.57)											
> 4		20		0.95 (0.86–1.04)	0.79		0.43 (0.25–0.61)	0.90										
**a***p*-Value test for trend. **b***p*-Value, one-way analysis of variance.																		

*Association of levels of exposure to inhalable metals with histone modifications.* We evaluated whether levels of H3K4me2 and H3K9ac were associated with the levels of personal exposure to metals in inhalable PM, as well as to PM mass, in both simple regression models and multivariable models adjusted for age, BMI, education, pack-years, and percent granulocytes. Results from unadjusted and adjusted models showed similar results (Table 4). In adjusted models, H3K4me2 increased in association with nickel, arsenic, and iron. H3K4me2 was not associated with aluminum, manganese, zinc, lead, PM_10_, or PM_1_ exposure levels in unadjusted or adjusted regression models (Table 4). H3K9ac was positively but not significantly associated with nickel and iron levels. H3K9ac was not associated with the levels of the remaining metals, PM_10_ or PM_1_ (Table 4).

**Table 4:** Association of personal level of exposure to PM metal components and total PM mass with H3K4me2 and H3K9ac.

		Association with H3K4me2								Association with H3K9ac						
		Unadjusted				Adjusted*a*				Unadjusted				Adjusted*a*		
Exposure		β-Coefficient (95% CI)		*p*-Value		β-Coefficient (95% CI)		*p*-Value		β-Coefficient (95% CI)		*p*-Value		β-Coefficient (95% CI)		*p*-Value
Aluminium		0.01 (–0.02 to 0.04)		0.36		0.02 (–0.01 to 0.05)		0.24		–0.04 (–0.11 to 0.02)		0.15		–0.03 (–0.1 to 0.03)		0.33
Manganese		0.02 (–0.01 to 0.04)		0.21		0.02 (–0.01 to 0.05)		0.17		–0.03 (–0.08 to 0.02)		0.25		–0.01 (–0.06 to 0.04)		0.73
Nickel		0.15 (0.03 to 0.28)		0.02		0.16 (0.03 to 0.3)		0.02		0.22 (–0.04 to 0.48)		0.10		0.24 (–0.02 to 0.51)		0.07
Zinc		0.05 (–0.01 to 0.11)		0.08		0.05 (–0.01 to 0.12)		0.12		–0.05 (–0.18 to 0.07)		0.41		–0.06 (–0.19 to 0.07)		0.38
Arsenic		0.16 (0.03 to 0.28)		0.02		0.16 (0.02 to 0.3)		0.02		0.18 (–0.08 to 0.45)		0.17		0.21 (–0.06 to 0.48)		0.13
Lead		0.02 (–0.01 to 0.04)		0.19		0.02 (–0.01 to 0.05)		0.16		–0.04 (–0.09 to 0.02)		0.16		–0.02 (–0.08 to 0.04)		0.52
Iron		0.12 (0.01 to 0.24)		0.04		0.14 (0.01 to 0.26)		0.03		0.21 (–0.02 to 0.45)		0.08		0.22 (–0.03 to 0.47)		0.08
PM_10_		0.03 (–0.03 to 0.09)		0.34		0.04 (–0.03 to 0.11)		0.23		–0.08 (–0.2 to 0.04)		0.17		–0.04 (–0.17 to 0.09)		0.51
PM_1_		0.02 (–0.06 to 0.1)		0.64		0.03 (–0.06 to 0.13)		0.45		–0.04 (–0.2 to 0.13)		0.65		0.00 (–0.18 to 0.18)		0.97
Data are regression coefficient (β) and 95% CI expressing the change in histone modifications (OD units) associated with an increase in exposure equal to the difference between the 90th and 10th percentile of the exposure distribution. **a**Multivariable regression models adjusted for age, BMI, education, pack-years, and percent granulocytes.																

*Association of cumulative exposure to inhalable metals with histone modifications.* Table 5 shows the associations of H3K4me2 and H3K9ac with cumulative exposures, estimated as the product of level of exposure to PM metal components or total PM mass and years of employment. Again, unadjusted and adjusted models showed similar results. In adjusted models, H3K4me2 increased in association with cumulative exposure to nickel and arsenic. H3K9ac showed significant associations with cumulative exposure to nickel and arsenic. Cumulative exposure to iron was positively but not significantly associated with both H3K4me2 and H3K9ac.

**Table 5:** Association of cumulative level of exposure*a* to PM metal components and total PM mass with H3K4me2 and H3K9ac.

		Association with H3K4me2								Association with H3K9ac						
		Unadjusted				Adjusted*b*				Unadjusted				Adjusted*b*		
Exposure		β-Coefficient (95% CI)		*p*-Value		β-Coefficient (95% CI)		*p*-Value		β-Coefficient**(95% CI)		*p*-Value		β-Coefficient**(95% CI)		*p*-Value
Aluminium		0.01 (–0.03 to 0.04)		0.61		0.02 (–0.02 to 0.06)		0.44		–0.02 (–0.09 to 0.04)		0.48		–0.03 (–0.1 to 0.05)		0.48
Manganese		0.01 (–0.02 to 0.04)		0.33		0.02 (–0.02 to 0.05)		0.35		–0.01 (–0.07 to 0.04)		0.63		0.00 (–0.06 to 0.05)		0.86
Nickel		0.11 (–0.01 to 0.23)		0.07		0.16 (0.01 to 0.3)		0.03		0.26 (0.04 to 0.49)		0.02		0.27 (0.01 to 0.54)		0.04
Zinc		0.06 (–0.03 to 0.15)		0.21		0.06 (–0.05 to 0.17)		0.30		–0.02 (–0.21 to 0.16)		0.80		–0.03 (–0.24 to 0.18)		0.78
Arsenic		0.14 (0.03 to 0.26)		0.01		0.16 (0.03 to 0.29)		0.02		0.30 (0.09 to 0.51)		0.01		0.28 (0.04 to 0.51)		0.02
Lead		0.02 (–0.02 to 0.06)		0.35		0.02 (–0.03 to 0.06)		0.38		–0.03 (–0.11 to 0.05)		0.42		–0.02 (–0.1 to 0.06)		0.65
Iron		0.09 (–0.03 to 0.21)		0.14		0.12 (–0.02 to 0.27)		0.09		0.25 (0.03 to 0.48)		0.03		0.24 (–0.03 to 0.5)		0.08
PM_10_		0.03 (–0.04 to 0.11)		0.41		0.05 (–0.04 to 0.14)		0.26		0.00 (–0.14 to 0.15)		0.99		0.00 (–0.17 to 0.17)		0.97
PM_1_		0.03 (–0.07 to 0.13)		0.53		0.07 (–0.06 to 0.21)		0.29		0.08 (–0.11 to 0.28)		0.40		0.08 (–0.17 to 0.32)		0.54
Data are regression coefficient (β) and 95% CI expressing the change in histone modifications (OD units) associated with an increase in exposure equal to the difference between the 90th and 10th percentile of the exposure distribution. **a**Cumulative levels of exposure were estimated as the product of personal level of exposure to PM metal components or total PM mass and years of employment in the job. **b**Multivariable regression models adjusted for age, BMI, education, pack-years, and percent granulocytes.																

## Discussion

The present study, based on a healthy worker population from a steel plant near Brescia, Italy, showed that exposure to some metal components of PM was associated with increased activating histone modifications measured in blood leukocyte samples. In particular, we found that both H3K4me2 and H3K9ac were higher in individuals with more years of employment in the plant and higher estimated cumulative exposures to arsenic and nickel.

To the best of our knowledge, this is the first study showing associations between metal exposures, such as nickel, arsenic, and iron, and histone modifications in human subjects. Our study was based on measures of histone modifications in blood leukocyte samples from healthy subjects, thus suggesting that exposure-related alterations of histone modifications may occur in normal tissues and possibly anticipate the onset of disease. Estimated effects of metal exposures on H3K4me2 in blood leukocytes were consistent with previous *in vitro* toxicology studies that showed that carcinogenic metals increased H3K4me2 in A549 human lung carcinoma cells (Sun et al. 2009; Zhou X et al. 2009). In particular, nickel and arsenic, which were most consistently associated with increased H3K4me2 in our analyses, were also previously found to increase H3K4me2 *in vitro* (Zhou X et al. 2009). In our study, we found that H3K4me2 was correlated with the levels of exposure to nickel, arsenic, and iron, but only nickel and arsenic showed significant associations with H3K4me2 when we evaluated cumulative exposures. Also, we found significant associations of cumulative exposures to nickel and arsenic with increased H3K9ac.

The changes observed in previous *in vitro* studies on lung carcinoma cells (Sun et al. 2009; Zhou X et al. 2009) and in our study in blood leukocyte samples suggest that induction of gene-activating histone modifications such as H3K4me2 and H3K9ac might be a systemic process detectable across different tissues. However, whether H3K4me2 and H3K9ac are induced in human lung tissues exposed *in vivo* to carcinogenic metals needs to be confirmed in future investigations. Metal components of inhaled PM have been shown to induce oxidative stress and inflammatory processes, which are known to affect histone modifications (Donaldson et al. 2003; Gilmour et al. 2003) and might specifically affect blood leukocyte measures. A series of experimental studies using ambient PM_10_ collected in Utah Valley (USA) near a local steel plant demonstrated that anthropogenic PM containing bioavailable transition metals have heightened acute inflammatory effects, and that PM oxidant generation ability was enhanced in PM with higher metal content (Dye et al. 2001; Frampton et al. 1999; Ghio and Devlin 2001). Previous studies have shown that exposures to PM or related airborne pollutants are associated with changes in proinflammatory and cancer-related gene expression in blood leukocytes (Feinberg and Tycko 2004; Franklin et al. 2008; MacNee and Donaldson 2003; Salnikow and Zhitkovich 2008) and with markers of gene expression control such as DNA methylation (Baccarelli et al. 2009; Bollati et al. 2007; Pavanello et al. 2009; Tarantini et al. 2009).

We investigated a population with well-characterized exposure that allowed us to compare subjects over a wide range of different exposure levels. We controlled for several potential confounders by fitting multivariable models that included several individual characteristics as independent variables. However, we cannot exclude the possibility that other unmeasured exposures that are present in foundry facilities, such as heat, carbon monoxide, and nonionizing radiation (Gomes et al. 2002; Lewis et al. 1992), might have influenced H3K4me2 and H3K9ac. In univariate analyses, we found that H3K9ac was positively associated with the education level of the study subjects. Although our study did not provide information to evaluate the biological basis for this association, our results showed that adjusted and unadjusted estimates for metal effects on histone modifications were remarkably similar, so confounding from education and other variables included in our models is unlikely.

Because of the limited number of study subjects, it is possible that the associations observed were attributable to chance. However, the occupational exposure and relatively controlled environment of a foundry provide a good setting for evaluating these mechanistic questions and limit bias and chance findings. Our study was based on subjects working in several work areas of the same factory and did not include a different population of subjects without a specific condition of exposure to inhaled pollutants. Limiting our investigation to individuals who have all been working in the same work facility avoided potential concerns related to the selection of external referents who might have differed from the exposed population in terms of socioeconomic factors and other characteristics determining hiring into the plant (Pearce et al. 2007). Nonetheless, the differences in the personal levels of exposure in our study group were large, providing sufficient contrast for identifying exposure-related changes in histone modifications. For example, the lowest level of PM_10_ observed in our study population (73.72 μg/m^3^) was only marginally higher than ambient PM_10_ levels measured in the geographic area in which the plant is located (average annual ambient PM_10_ levels between 41 and 57 μg/m^3^ were recorded in the year of the study by different ambient monitoring stations in Brescia area) (Anselmi and Patelli 2006), whereas the highest level was 1220.17 μg/m^3^. The subjects with the highest exposures to arsenic and nickel had exposure levels that were 36 and 31 times higher, respectively, than those for the least-exposed subjects. Although we based our study on a group of steel workers with average exposures higher than that of the general population, the levels of exposure to metals in our study were all lower than the commonly accepted threshold limits for industrial settings (American Conference of Governmental Industrial Hygienists 2009).

Because we determined differences in exposures within our study population by the different tasks routinely performed by each of the study subjects, the personal exposure levels we measured in the week of the study reflected the usual exposure of the study subjects. We confirmed this by the high correlation (*r*^2^ > 0.90) between exposure measures repeated over 3 years in a subset of the study population. Employment records showed that all the subjects included in the present study performed the same tasks for all the years they had been employed in the plant, suggesting that contrasts of exposure within the study population might have remained stable over time. Therefore, we estimated long-term cumulative exposure as the product of the levels of exposure by years of employment in the plant. However, because most of the study subjects had worked for more than a decade in the plant, it is possible that changes in production or exposure protection regulations might have occurred over such an extended period of time. Therefore, we recognize that the estimated cumulative exposures we used in the present study are prone to exposure misclassification, and the results based on these metrics of exposure should be interpreted with caution.

## Conclusions

Our results suggest that exposure to some metal components of PM, including nickel and arsenic, increased the two genes activating H3K4me2 and H3K9ac in blood leukocytes collected from healthy steel workers. Changes in the genomic levels of histone modifications may affect gene expression and contribute to the carcinogenic properties of inhalable nickel and arsenic. Further studies are required to directly link these changes with exposure-related increases in the risk of cancer, as well as to identify specific genes and pathways that are affected by the exposure-related changes in histone modifications.
